# The potential of neurofilaments analysis using dry-blood and plasma spots

**DOI:** 10.1038/s41598-019-54310-y

**Published:** 2020-01-09

**Authors:** Vittoria Lombardi, Daniele Carassiti, Gavin Giovannoni, Ching-Hua Lu, Rocco Adiutori, Andrea Malaspina

**Affiliations:** 1 Blizard Institute, Queen Mary, University of London, London, UK; 2 School of Medicine, China Medical University, Taichung, Taiwan

**Keywords:** Amyotrophic lateral sclerosis, Prognostic markers

## Abstract

The lack of biomarkers for an early diagnosis of neurodegenerative disorders (NDs) has hampered the development of therapeutics whose effect would be enhanced by a timely intervention. Neurofilaments light chain (Nf-L), an integral part of the axonal structure, has emerged as a robust fluid biomarker for fatal neurodegenerative disorders like amyotrophic lateral sclerosis (ALS). To facilitate large-scale studies into early-stage neurodegeneration, reduce costs of samples collection/processing and cold-chain storage, we describe the measurement of Nf-L in blood fractions obtained from dry blood spots (DBS) and dry plasma spots (DPS), two filter paper-based remote blood collection tools. To test the feasibility of using this approach, Nf-L analysis in DBS/DPS is compared to that in plasma obtained from the same blood sample, looking at Nf-L discriminatory power in the clinical stratification of ALS compared to healthy controls. With the best pre-analytical treatment for total protein recovery and using highly sensitive immunoassays, we report the detection of different Nf-L levels in DBS elute compared to reference plasma and DPS from the same blood samples. However, Nf-L measurement in DBS elutes provides a very good discrimination of ALS from healthy controls which is comparable to that obtained using plasma Nf-L assays. With the available immunodetection methods, we show that Nf-L measurement based on DPS microsampling is similar to that in plasma. The filter-paper biophysical characteristics and the interference of high haemoglobin concentration released by erythrocyte lysis is likely to perturb Nf-L detection in DBS elute. Further studies into DBS-based Nf-L detection and its analytical optimization are needed to make this method suitable for routine Nf-L blood analyses in neurodegeneration.

## Introduction

The rise of neurodegenerative disorders (NDs) in the ageing population is increasing demand on already stretched Health Services and is a major financial burden for the society^1^. The development of tools for early diagnosis allowing timely therapeutic interventions is one of the responses to this problem^2^. To this end, it is important to acquire easily measurable, cost-effective and sensitive biomarkers of axonal loss, the process underpinning neurodegeneration, which can also be used as surrogate markers of treatment response in clinical trials. The design and the implementation of effective clinical trials can also be improved by better means of biomarkers-based clinical stratification. Biological predictors of rate of disease progression, for example, will reduce uncertainties surrounding the true disease modifying effect of a medication in clinically heterogeneous diseases.

The measurement in blood and cerebro-spinal fluid (CSF) of neurofilaments (Nf), the building blocks of neurons and axons, is increasingly seen as a breakthrough in the development of a *universal* biomarker of neurodegeneration and potentially useful also to study ageing and environmental exposure like trauma^3,4^. CSF, the main repository of by-products of neuronal destruction, is enriched in Nf and unlike other peptides linked to neurodegeneration such as Tau and Amyloid beta, Nf expression in CSF is highly correlated to that in blood^5,6^. As serial lumbar punctures to procure CSF for biomarkers analysis may be impractical in advanced and uncooperative patients and disease monitoring by structural and functional brain imaging is expensive and logistically complex^7^, blood may represent the ideal source to track any meaningful disease signal of neurodegeneration cost-effectively in large biomarkers studies and clinical trials. Both light and heavy neurofilament chain (Nf-L; Nf-H) measurement in blood is now possible thanks to highly sensitive immunodetection platforms like Meso Scale Discovery (MSD) and single molecular array (Simoa), which provide readings at picogram and femptogram levels respectively^3,8–10^. Therefore, the role of Nf as biomarkers of neurodegeneration can only be fully appreciated through a large-scale survey of their expression at a population level, factoring variables including age, ethnic origin as well as the association of relevant co-morbidities.

To enable large-scale biomarker studies, the costs of sensitive analytical platforms and of properly funded and staffed services are considerable. Budgets include patients attendance to busy outpatient departments, phlebotomy, sample processing and storage at high energy-consuming super-low temperatures^11^. High-costs and low energy efficiency have pushed alternative means of samples collection and storage ensuring a similar analytical performance as traditional forms of sampling, but with no or less requirements for sample processing. Dried plasma spot (DPS) and dried blood spot (DBS) on filter paper can be a remote, quick and inexpensive way of obtaining blood microsamples for the measurement of a large number of analytes and their use may be a feasible, non-expensive and energy-saving means of disease screening and treatment monitoring in non-hospitalized, public health settings^12,13^. The use of DBS and DPS has no requirements in terms of cold chain for transport and storage. Plasma and serum obtained from processed blood can be directly spotted on to absorbent paper, stored at room temperature, and later reconstituted with a simple elution step. Recently, a new concept of plasma prep cards (Noviplex™) has become available. This next generation DBS system makes it possible, after spotting blood drops onto a card, to physically remove blood cells which are compartmentalized in the superficial layer of the card. The result is a stable DPS which delivers up to 7.5 µl plasma, which can be used to generate analytical results that better agree with plasma analysis by conventional methods (https://www.novilytic.com/noviplex-plasma-prep-cards/). Protocols for the use of DBS and DPS to measure a range of analytes for diagnostic purposes like human immunodeficiency virus, Cytomegalovirus, Mycobacterium Tuberculosis, Leishmania and to test the detection efficiency of low abundance analytes like cytokines are now available^10–17^. Analytical performance for a number of target molecules in DBS and matched plasma/serum samples may differ as a measure of how the filter matrix modifies the relative composition of the blood components in the final product of elution.

Here we have used highly sensitive immunodetection assays^9,14–16^ to test neurofilament light chain (Nf-L) expression in elute from Protein Saver 903 cards DBS and from DPS stored at ambient temperature and compared these to standard Nf-L plasma measurement. Taking advantage of red cells, plasma and DBS extracted from the same blood samples from healthy controls and patients with amyotrophic lateral sclerosis (ALS), a clinically heterogeneous and invariably fatal neurodegenerative disorder, we have looked at whether DBS-based Nf-L measurements maintain the same discriminatory power reported using plasma assays where Nf-L differentiates healthy from affected individuals as well as fast versus slow progressing ALS patients. We show that DBS Nf-L reading is different from that obtained in matched plasma, although DBS Nf-L detection enables a good separation of ALS from healthy controls (HC), similarly to matched plasma Nf-L levels. The differences in Nf-L detection is likely to be due to haemoglobin interfering with Nf-L detection in the haemolytic elute from the saver card. Elute from DBS presents a different proteolytic profile for Nf-L when compared to matched plasma which may explain the different patterns of immunodetection. Finally, differences between Nf-L detection in DBS elute and matched plasma are due to the high dilution of DBS products of elution compared to the highly concentrated proteinaceous mix in plasma, after removal of the blood corpuscular fraction. The study provides the basis to further explore the remote collection of blood micro-samples immobilized on dry filter paper for an efficient and cost-effective use of Nf analysis in large-scale biomarkers studies.

## Results

### Patients and samples selection

17 Healthy Controls (HC) with no medical history of disorders known to affect Nf levels like mechanical injuries, ischaemic or inflammatory pathologies of the central nervous system or peripheral neuropathies (male/female ratio: 6/11; average age at sampling: 58 (±7)) along with 5 neurological controls (NC, male/female ratio: 3/2; average age at sampling: 70 (±11)) including congenital peripheral neuropathy, myasthenia gravis, frontotemporal dementia, progressive muscle atrophy and parkinsonism) and 20 age/gender-matched individuals with a diagnosis of amyotrophic lateral sclerosis (El Escorial diagnostic criteria^17^) were included in the study. ALS patients were sub-grouped in fast (ALS-Fast, average age at sampling: 61(±13)) and slow (ALS-Slow, average age at sampling: 70 (±10)) progressing individuals based on progression rate to last visit (PRL: ALS-Slow < 0.6 and ALS-Fast > 0.9). Demographic and clinical characteristics are reported in Table 1. 30 ml blood was drawn by venepuncture from each participant to obtain plasma and serum and between 0.7 to 1 ml was spotted on to 2 protein saver cards (PSC), one stored at room temperature and one at −80 °C. To obtain dry plasma spots (DPS), we either spotted blood drops on to a Noviplex cards (a multi-layered card system supporting a simple two-step recovery of plasma spots; https://www.biocompare.com/23540-Blood-and-Tissue-Products/5017746-Noviplex-Cards-Dried-Plasma-Spot-Technology/) or spotted processed plasma (and serum) directly onto a PSC.

**Table 1:** Summary of clinical/demographic information and of the biochemical markers tested in the study participants.

Participants	n	Age y (SD)	Sex M/F	Hb g/L (SEM)	Haematocrit % (SEM)	ALS-FRS (SEM)	Progression rate (SEM)	Nf-L pg/ml (SEM)					
								MSD Plasma	MSD DBS	SIMOA Plasma*	SIMOA DBS*	SIMOA Plasma	SIMOA Np-DPS
HC	17	58 (±7)	6/11	140 (±3.6) n.12	0.4 (±0.008) n.12	—	—	21.41 (±2.15)	17.49 (±6.34)	1.74 (±0.28)	0.46 (±0.07)	23.19 (±16.5)	1.03 (±0.67)
Neur-Ctr	5	70 (±11)	3/2	—	—	—	—	—	—	—	—	15.68 (±3.4)	0.60 (±0.14)
ALS-Slow	9	70 (±10)	6/3	146 (±6) n.7	0.4 (±0.01) n.7	43 (±1)	0.21 (±0.08)	131.52 (±40.2)	47.90 (±15.0)	4.14 (±0.78)	3.10 (±1.06)	—	—
ALS-Fast	11	61 (±13)	4/7	139 (±5) n.8	0.4 (±0.01) n.8	31 (±1)	1.87 (±0.21)	194.41 (±46.4)	34.44 (±12.5)	26.62 (±5.76)	9.02 (±1.93)	—	—
ALS**	5	76 (±3)	2/3	n/a	n/a	n/a	n/a	—	—	—	—	43.89 (±16.3)	1.33 (±0.47)
Correlation ***	—	—	—	—	—	—	—	Plasma/DBS (1) P = 0.2, R^2^ = 0.09		Plasma/DBS (2) P < 0.0001, R^2^ = 0.7		Plasma/Np-DPS (3) p < 0.0001, R^2^ 0.9	

The level of neurological impairment is represented by the ALS functional rating scale-revised (ALSFRSr), comprising a scale of 1 to 48, with lower scores corresponding to a higher degree of neurological impairment. ALS patients are grouped based on the rate of disease progression including fast-progressing (ALS-Fast) and slow-progressing (ALS-Slow). Progression rate at last visit (PRL) is calculated as 48 (approximation at disease onset) minus the ALSFRSr score at last visit divided per the number of months from disease onset to last visit. For the purpose of this study, ALS-Fast is considered as PRL > 0.9 and ALS-Slow PRL < 0.6. Nf-L levels were measured using the mesoscale discovery system (MDS) and single molecular array (Simoa). *Equal loading protein concentration (10 µg/µl). **Additional ALS patients whose matched plasma and Np-DBS have been tested by Simoa for Nf-L. ***Linear correlation analysis of Nf-L levels between matched plasma and DBS elutes, tested using MSD (1), Simoa (2) and linear correlation analysis of Nf-L levels between matched plasma and Np-DPS elutes tested on Simoa (3). HC: Healthy Control. Neur-Ctr: Neurological controls. ALS: amyotrophic lateral sclerosis.

### Elution conditions and total protein concentration on elution

**Figure 1:**
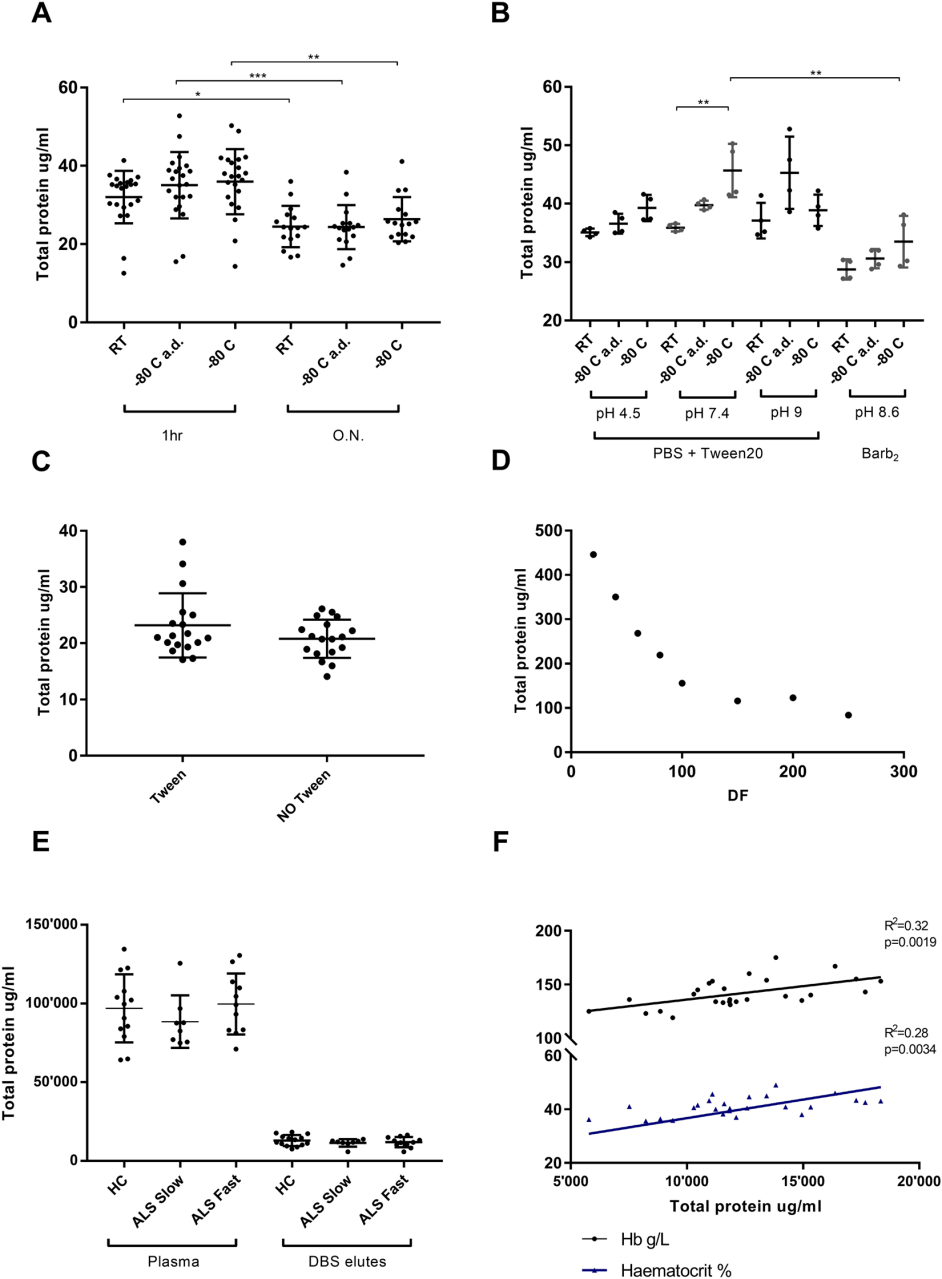
Analysis of DBS elution conditions. (**A**) Total protein concentration in elutes obtained from DBS stored at room temperature, at −80 °C and at −80 °C and air dried (a. d.) for 10 minutes before elution. Elution is performed with buffer for 1 hour at 37 °C or overnight (ON) at 4 °C. There is no variation of total protein concentration in elutes obtained from DBS kept at different storage conditions but there is a significant reduction of total protein concentration when the elution is undertaken ON at 4 °C (Bradford measurement, multiple comparisons Kruskal-Wallis test; RT p = 0.03, −80 °C a.d. p = 0.0006, −80 °C p = 0.002); (**B**) total protein content in elutes according to pH, storage conditions and use of Tween20 or Barb2. Elution with PBS + Tween20 at 7.4 pH of air-dried DBS stored at −80 °C has the best yield and less variability of total protein concentration in elution products (Bradford measurement; multiple comparisons Kruskal-Wallis test, Tween20 RT vs −80 °C p = 0.007, Tween20 vs Barb_2_ p = 0.008); (**C**) no changes in total protein concentration (Mann-Whitney test, p = 0.33) when elution is performed with or without Tween in the buffer (Pierce measurement). (**D**) Increasing volumes of elution buffer show linearity in total protein concentration in the elutes up to 1/100; linearity is lost for higher elution volumes; (**E**) total protein concentration (Pierce measurement) in DBS elutes from Healthy Controls (HC), ALS-Fast and ALS-Slow DBS is about 8 to 10 fold lower than total protein concentration measured in matched plasma samples. There are no differences in the DBS protein levels between HC and ALS patients. (**F**) Significant correlation between Haemoglobin (Hb), Haematocrit (Hct) and total protein concentrations measured in DBS obtained from the same blood samples (p = 0.0019, R^2^ = 0.32 and p = 0.0055, R^2^ = 0.28 respectively; linear regression analysis). DF: dilution factor.

Total proteins concentration was tested using Pierce (Pierce™ BCA Protein Assay Kit - cat. 23225) and Bradford methods (Bio-Rad Protein Assay Kit II Bio-Rad - cat. 5000002) to evaluate elution conditions for DBS stored at room temperature (RT) or at −80 °C, using matched plasma samples as reference (Fig. 1). A subset of DBS kept at -80 were air dried (a.d.) for 10 minutes before elution. Volume, pH and presence of a protease inhibitor were the eluent conditions tested and DBS were kept at room temperature (RT) for one hour or overnight (ON) at 4 °C on elution. In DBS from a group of 22 controls, storage conditions (RT vs −80 °C) after blood collection (with or without a.d. before elution) did not affect protein concentration (Fig. 1A). However, ON elution at 4 °C resulted in a significant reduction of total protein content compared to 1-hour elution at 37 °C (Fig. 1A). The buffer pH (range of 4.5–7.4) changed the total protein content in the elutes and a.d. treatment of −80 °C stored cards showed a higher and more consistent protein concentration (Fig. 1B). The presence of Tween20 in the eluent did not modify the yield of total proteins in the products of elution (Fig. 1C).

With increasing volumes of elution buffer, there was linearity of total protein concentration in the final elute with up to a 1/100 dilution factor. Linearity was lost for higher elution factors (Fig. 1D). Total protein content measured 24-hour after elution did not differ significantly if proteases inhibitor (PI) was added to the elution buffer or not (Supplementary Fig. 1). There were no differences in total protein levels in DBS elutes and in matched plasma samples comparing participant sub-groups including ALS (ALS-Fast n: 11; ALS-Slow n: 9) and Healthy Controls (HC n: 14), suggesting that the biological differences between ALS sufferers and healthy individuals did not impact in the overall protein concentration in the elutes from DBS. Average total protein concentration was 7 to 8 folds higher in plasma samples compared to matched DBS elutes (Fig. 1E). Furthermore, total protein levels of the elutes from DBS of the three subgroups appeared in a much tighter range (smaller standard deviations) compared to the variability and wider range of concentrations observed in total protein levels detected in the matched plasma samples (Fig. 1E). No significant correlation between DBS and matched plasma samples total protein levels was found (Supplementary Fig. 2). As previously reported in the literature^18^, protein recovery from DBS did not change using different detergents (Fig. 1B,D).

These findings indicate that the physical properties of the protein saver card (PSC) dry paper may condition both absorption of blood and release of proteins, a process which may be independent from the amount of total proteins which maintains a linear relation to the volume of eluent (lost above 100 µl dilution, Fig. 1D).

Total protein levels in DBS elutes positively correlated with the source blood samples haematocrit (p = 0.0019) and haemoglobin (p = 0.0055) levels (Fig. 1F), while no correlation was found between plasma total proteins and haematocrit/haemoglobin (Supplementary Fig. 3). This may be explained by the so-called *chromatographic effect* previously described by Denniff, P *et al*.^19^, which defines how the retention of whole blood is affected by haematocrit and by the physical characteristics of PSC. The reported linear and inverse correlation between spot area and blood haematocrit at a given volume of blood (the higher the haematocrit value the smaller the blood spot and vice versa) may influence the relative concentration of haemoglobin and also of total proteins eluted from the fixed (punched) area of the DBS, which in our case is 6 mm in diameter (Fig. 1F). Haematocrit is also known to introduce a bias in the detection of most analytes in DBS elutes^20,21^.

Based on the above results, to test total proteins and Nf-L in elution products, we have utilised DBS stored at RT treated with 60 µl of elution buffer for 1-hour at 37 °C.

### Qualitative analysis of products of elution from dry spots and reference plasma samples by gel separation

**Figure 2:**
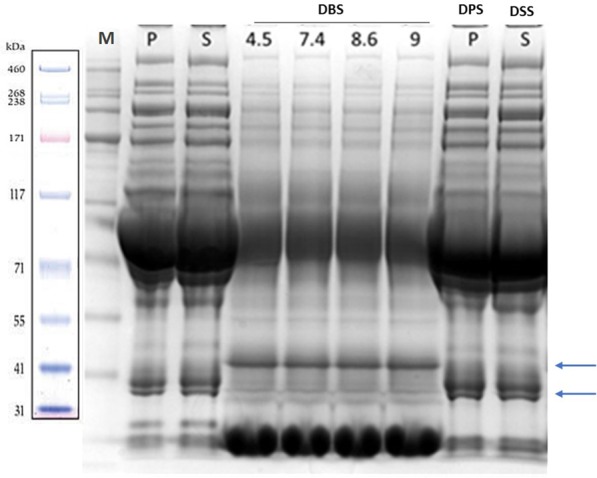
Example of 4–12% Comassie gel resolution of plasma (P), serum (S), dry blood spots (DBS) eluted at PH 4,5, -7,4, -8,6 and 9, dry plasma spot (DPS) and products of elution from protein saver cards spotted with serum (dry serum spot – DSS) obtained from the same individual’ blood sample. Plasma and serum were diluted 1:100 at room temperature (RT) while DBS were diluted 1:60 after 1 h at 37 °C. The same protein concentration was loaded in each lane for all samples (100 micrograms/lane). Gel bands from P, S and from DPS and DSS appear equivalent. Arrows indicate DBS bands which are different from those obtained from P, S, DPS and DSS, particularly at low molecular weight (red cell lysis products). The bottom large and intense bands in the DBS lanes are likely to be products of haemolysis. The most prominent band just above 71 kDa is albumin. The high concentration of albumin is likely to affect electrophoretic migration, skewing the correspondent band position (with respect to the protein marker, M) slightly above the expected molecular weight (66 kDa). M: protein marker.

SDS Page gel electrophoresis and Western blot analysis were performed to resolve plasma and serum fractions and compare them to the elutes from DBS, DPS and from filter paper spotted with serum (dry serum spot; DSS; Fig. 2) obtained from the same blood samples. 100 micrograms of total protein obtained from DBS/DPS/DSS elutes and from plasma/serum samples were loaded in a Nu-Page 4–12% Bis-Tris gradient gel. Visual inspection of the gels revealed equivalent band profiles when plasma was compared to DPS and serum to DSS (Fig. 2), suggesting that the elution from filter paper spotted with serum and plasma generated fluids that are indistinguishable from plasma and serum obtained using standard blood processing. Densitometric analysis confirmed similar concentrations for plasma, serum and related DPS/DSS (data not shown). DBS elutes electrophoresis revealed similar but fainter bands when compared to plasma and serum (and related DPS, DSS), while additional large bands were visible at lower molecular weight likely to represent products of haemolysis (Fig. 2). Banding intensity did not change with the chemical conditions of elution, including pH and presence of Tween 20 (data not shown).

### Neurofilaments analysis by Meso Scale Discovery (MDS) and single molecular array (Simoa)

Nf-L analysis was performed in blood samples from 20 Slow/fast ALS and 22 controls (including 17 healthy and 5 neurological controls) using MSD and Simoa platforms, the two most sensitive immunodetection platform for Nf-L analysis currently available^9^. The choice of these cohorts was based on previously published data demonstrating that plasma Nf-L provides a robust means of stratification of ALS between fast and slow progressing individuals and separation between ALS and controls, including healthy individuals and those with other neurological conditions^3,6^. Our goal was to ascertain whether Nf-L analysis in blood products obtained from non-conventional DBS or DPS storage methods reproduced the same readings obtained from plasma and had the same discrimination power when comparing ALS to controls, or stratifying ALS according to rate of disease progression. Individuals with a neurological disease other than ALS were added to the control mix in order to include samples expected to have higher levels of Nf-L and test sensitivity and specificity for ALS diagnosis in plasma and DBS-derived samples.

Given the reported filter paper and haemoglobin effects in determining the protein concentration in the final products of DBS elution^19^, Nf-L immunodetection was performed using equivalent loading volumes normalised to each sample haematocrit (Hct) levels (1^st^ experiment: MSD platform) and equivalent protein concentrations (2^nd^ experiment: Simoa platform) as reported below.

**Figure 3:**
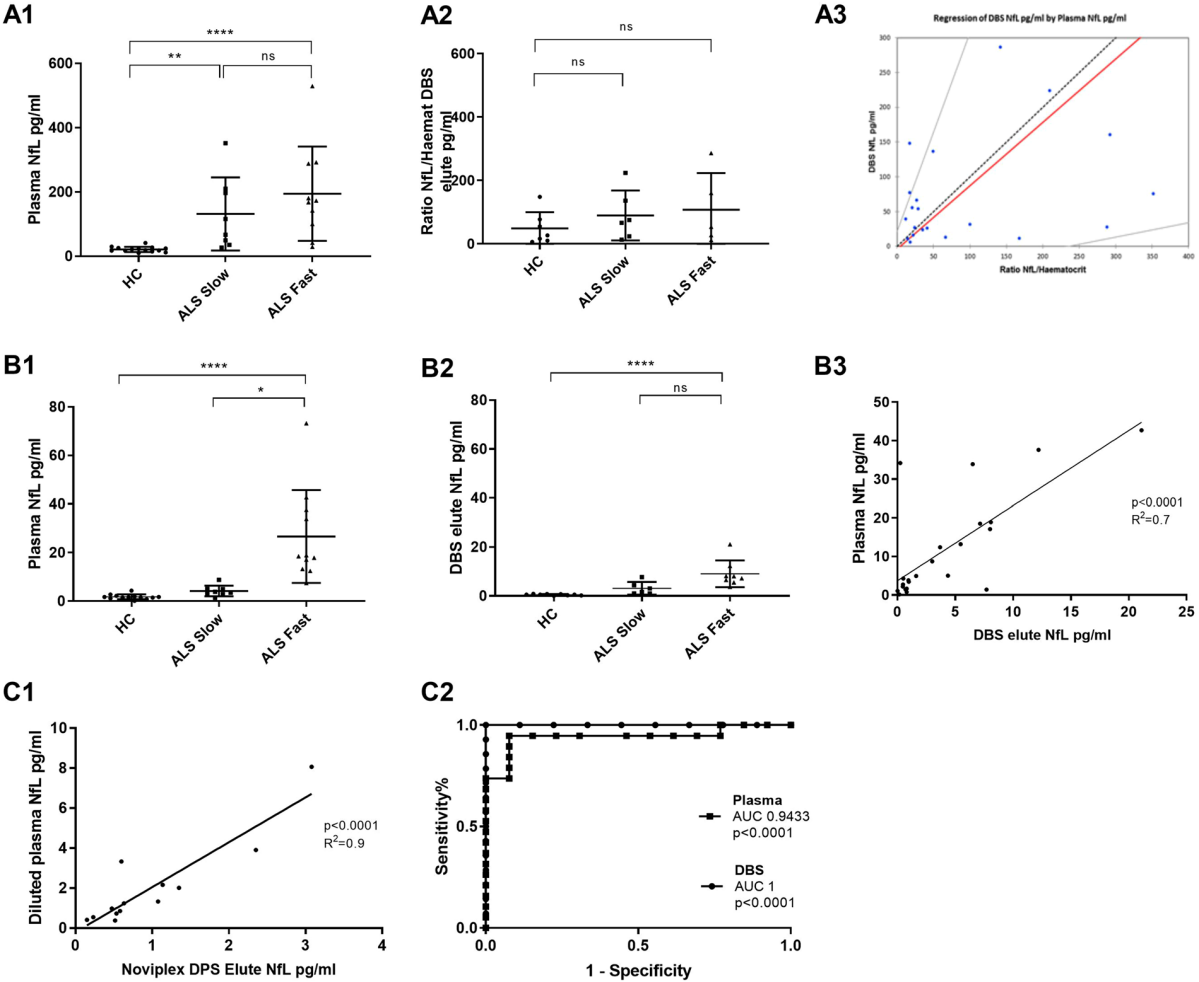
Nf-L analysis by MSD and Simoa in plasma samples, DBS and DPS elutes. A.1, A.2: Nf-L measurement using MSD in plasma and DBS elutes with equal volume loading. There is a trend of Nf-L up-regulation in DBS elutes from ALS-Fast compared to ALS-Slow and HC which does not reach statistical significance, while Nf-L analysis in matched plasma samples shows the expected statistically significant up-regulation of Nf-L in ALS-Fast compared to ALS-Slow and HC (multiple comparisons Kruskal-Wallis test: HC vs ALS-Slow p = 0.002; HC vs ALS-Fast p < 0.0001). In DBS elutes from controls, three values were below the low limit detection (<6 pg/ml: 3.6, 4.8 and 2.4 pg/ml). A.3, Passing–Bablok regression statistical analysis showing a modest correlation between plasma Nf-L levels and matched DBS elute Nf-L/Haematocrit ratios (intercept interval includes 1 and the slope coefficient includes 0). B.1, B.2: Nf-L analysis in plasma and DBS elutes using equivalent loading concentration (10 µg/µl) performed using Simoa. Group analysis using Kruskal-Wallis test, demonstrated statistically significant upregulation of Nf-L in ALS-Fast compared to HC (p < 0.0001) in DBS elutes similarly to what shown for matched plasma samples (p < 0.0001). Differences between ALS-Fast and ALS-Slow are significant in plasma, but not in DBS. B.3: Linear regression analysis showing positive correlation between Nf-L levels obtained from plasma samples and from DBS elutes (Simoa; p < 0.0001, R^2^ = 0.7). C.1: Positive correlation between Nf-L levels in elutes from dry plasma spots (Noviplex cards, Np-DPS) and matched plasma samples using a linear regression analysis, (p < 0.0001, R^2^ 0.9). DBS: dry blood spots, DPS: dry plasma spots, MSD: meso-scale discovery, Simoa: single molecular array. C.2: Receiver operating curve (ROC) analysis comparing plasma Nf-L and matched DBS elutes Nf-L for the separation of ALS individuals from controls (plasma: p < 0.0001, AUC: 0.9433; DBS elute: p < 0.0001, AUC: 1).

Nf-L measurement in plasma samples by MSD using the same starting volume showed the previously described up-regulation of Nf-L in ALS-Fast compared to ALS-Slow and HC in plasma^6^ (Fig. 3A.1), while in matched DBS elutes, there was a trend for higher expression in ALS-Fast compared to ALS-Slow and HC of the Nf-L/haematocrit ratio which did not reach statistical significance (Fig. 3A.2). A mild correlation between Nf-L levels in DBS elutes and matched plasma samples expression was identified when using the Passing–Bablok regression statistical analysis, where the Nf-L/haematocrit ratios were plotted on the x axis (Fig. 3A.3).

Next, to circumvent the bias of DBS elution and chromatographic effect due to haematocrit and DBS physical properties on total protein concentration, we have tested Nf-L in the same samples using a more sensitive platform like single molecular array (Simoa – Quanterix)^9^ and the same amount of total protein was loaded for DBS elutes and for plasma samples. Equal loading concentration and the use of Simoa resulted in a good separation of ALS-Fast from ALS-Slow and HC using DBS elutes, along with what seen with matched plasma samples (Fig. 3B.1,B.2). As previous studies on Nf-L fluid expression have identified a strong association with age (22) and considering the average age difference of our controls and ALS patient subsets (Table 1), the Nf-L group analyses (Fig. 3A1,B1) were also performed after age adjustment. There were no differences in the reported group separation after age adjustment (data not shown). Receiver operating characteristic (ROC) analysis was used to compare the performance of plasma Nf-L and matched DBS elutes Nf-L to disciminate ALS individuals from controls (plasma: p < 0.0001, AUC: 0,9433; DBS elute: p < 0.0001, AUC: 1; Fig. 3C.2). Separation of ALS from controls was achieved with high level of specificity and sensitivity using Nf-L levels from both plasma and matched DBS. Linear regression analysis of Nf-L expression values in plasma and DBS elutes showed a good correlation (Fig. 3B.3; p < 0.0001- R^2^ = 0.7). Normalization with haemoglobin or haematocrit of the Nf-L concentration in DBS elute did not improve the statistical significance and the separation in the group and ROC analyses (data not shown). In line with what observed in our total protein analysis in DBS elute, Nf-L expression levels were on average 2.5-fold lowers in DBS elutes compared to that in matched plasma samples, with a much tighter distribution around the mean compared to the variability seen in plasma Nf-L levels (Fig. 3B.1,B.2).

Nf-L levels measured in elutes from 13 DPS obtained using Noviplex cards microsampling (Np-DPS), inclusive of healthy controls as well neurological controls and ALS patients, showed a high correlation with Nf-L levels measured in the same volume of matched plasma samples (p < 0.0001; R2 0.9) (Fig. 3C.1). Both Np-DPS elutes and matched plasma samples were at the same dilution fold before analysis (Fig. 3C.1).

### Western blot analysis of Nf light chain (Nf-L) in plasma, DBS elute and red blood cells

**Figure 4:**
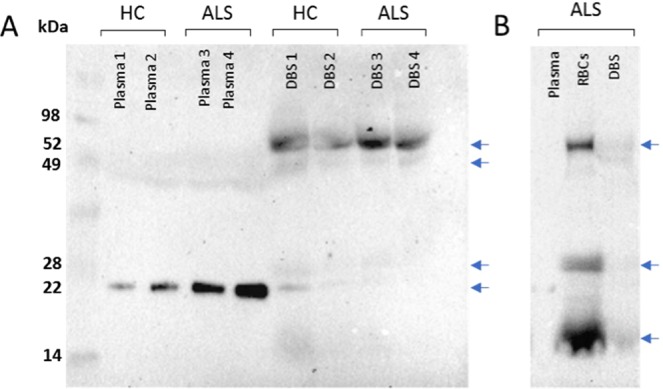
Western blot analysis of Nf-L expression in plasma, DBS elutes and RBCs obtained from the same blood samples. 100 µg of proteins from each blood component was loaded in a lane of a 10% gel. (**A)**, plasma Nf-L expression in Healthy Controls and ALS patients showing a band at 22 kDa while a 52 kDa band is visible in DBS elutes. 22 kDa bands intensity demonstrate higher Nf-L expression in plasma from ALS patients compared to Healthy Controls. (**B**) WB analysis of Nf-L comparing RBCs, DBS elutes and plasma from the same blood samples showing similar bands in RBCs and DBS. Plasma Nf-L appears at the same 22 kDa molecular weight level as reported in (**A**). The 22 kDa band in plasma shows a higher protein abundance in ALS patients compared to HC. The 52 kDa band in DBS does not appear different in ALS and HC. DBS: dry blood spots. RBCs: red blood cells. HC: Healthy Controls.

Western blot (WB) analysis with anti-Nf-L Ab identified Nf-L bands at different molecular weight in plasma and DBS elutes, in keeping with different Nf-L proteolytic profiles (Fig. 4A). In HC and ALS patients plasma, WB detected a 22 kDa Nf-L band with a significantly higher expression in ALS (Fig. 4C). In DBS elutes, Nf-L bands were detected at 52 (stronger intensity), 49, 28 and 22 kDa (lighter intensity; Fig. 4A). The 52 kDa bands in DBS did not significantly differentiate ALS patients from controls (Fig. 4D). To gather more information on the source of Nf-L in the DBS elute, we have repeated the same experiment comparing plasma to red blood cells lysate and peripheral blood mononuclear cell (PBMCs) extracted from the same HC and ALS patient’ samples. Nf-L bands in RBCs appeared at approximately the same MW levels as those detected in DBS elute (high intensity 52 kDa and 14 kDa bands with a fainter 28 kDa band). This finding strongly supports the presence of Nf-L in erythrocytes. No Nf-L bands were detected in PBMCs.

### Nf-L measurement in red blood cells by single molecular array (Simoa)

**Figure 5:**
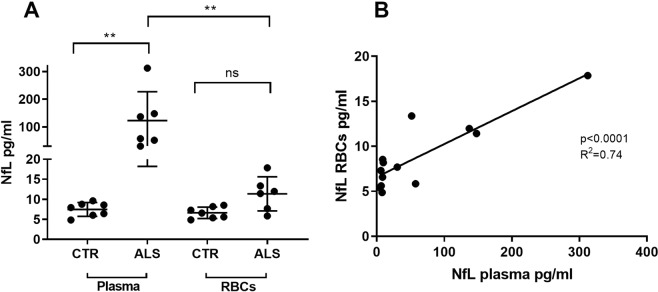
Nf-L analysis by Simoa in plasma and red blood cells (RBCs) from the same blood samples. (**A**) Nf-L analysis in plasma and RBCs lysates from ALS cases and HC demonstrates a statistically significant upregulation of Nf-L in plasma from ALS individuals compared to HC (Kruskal-Wallis test, p < 0.0022). Nf-L appears to be expressed in RBCs and over-expressed in ALS compared to HC. 5 (**B**) Positive correlation between Nf-L levels in matched plasma and RBCs lysates tested using Simoa (linear regression analysis, p < 0.0001, R^2^ = 0.74).

Nf-L measurement was performed in both plasma and red blood cells samples from n.6 Fast ALS and n.7 Healthy Controls (HC) using the Simoa platform (Fig. 5A). Nf-L appeared expressed in RBCs and compared to the Nf-L analysis in the matched plasma samples, it was (non-significantly) over-expressed in RBCs from ALS patients compared to HC (Fig. 5A). There was a significant correlation between the NF-L levels in plasma and in RBCs (r2 = 0.74 p < 0.0001; Fig. 5B).

## Discussion

In this study, we look at the measurement of Nf-L, an emerging biomarker of neurodegeneration, in microsamples of blood collected and stored in dried blood spots (DBS) and in plasma obtained from dry plasma spots (DPS). Using the most sensitive immunoassays currently available, we show that Nf-L measurement in products of elution from DBS is possible, but qualitatively and quantitatively different from determinations in plasma obtained using standard processing of the same blood samples DBS are derived from. This may be explained by the interference in the antibody-based analysis of high concentration of haemoglobin and of cell membrane proteins retained after elution of DBS, which are in a different dilution state compared to the highly concentrated extra-cellular protein mix found in processed plasma samples.

Nf-L detection in DBS elute is constrained by factors which are intrinsic to the physical interaction of whole blood with the saver card, whose effect on the release of protein upon dilution is far from being understood. Despite these neurochemical differences, Nf-L levels in DBS elutes provide a good level of discrimination between ALS and age-matched controls when Nf-L measurement in plasma is used as reference. It is nevertheless unclear to what extent Nf-L analysis using DBS elutes would be possible and by all means, informative, in clinical situations where Nf-L levels are expected to be much lower than the high blood concentration normally encountered under ALS pathological conditions and in the same age group control individuals. For example, the detection of minor changes in the lower picomolar or sub-picomolar range of Nf-L concentrations found in blood from newborn babies may not be possible, a problem that may limit considerably the use of DBS for Nf-L analysis in an area of medicine where difficult access to blood and the importance of subtle Nf-L changes linked to brain hypoxia, would see the use of DBS as an important development in routine clinical practice.

However, given the mounting evidence that Nf-L is an informative biomarker of axonal loss with a potential widespread use in clinical studies of neurodegeneration, ageing and post-natal brain injury, the importance of our preliminary and proof of principle data rests primarily on the fact that inexpensive remote collection, storage and transport of blood without the need of a cold-chain maintenance, could potentially be deployed in the development of large scale and population-based investigations into the bioavailability of Nf-L^22^.

Work is ongoing to broaden our knowledge on Nf expression in bio-fluids and establish how Nf levels change as a consequence of injury, remodelling and plasticity in affected brain tissue, or simply as a consequence of the progressive brain volume loss associated with ageing which is accelerated in neurodegenerative disorders^23–25^. To reduce costs associated with traditional blood sample collection, processing and storage, efforts to devise effective systems for remote blood collection and RT storage have multiplied^26^. Drops of blood for biochemical analysis can be collected as capillary (whole) blood after finger pricking, a minimally invasive alternative to venepuncture circumventing the need for phlebotomy and attendance to busy Outpatient Departments^13^. DBS and DPS can be safely kept at RT and posted to a reference lab without any further processing and storage at ultra-low temperature, as otherwise required for conventionally processed plasma and serum samples. The importance of the wide implementation in clinical trials of low-cost biomarkers is paramount: biomarkers help researchers identify and monitor different patient sub-groups, where the disease can be defined by specific molecular, genetic or immunological traits. This approach facilitates novel ‘adaptive designs’ of confirmatory trials, with inclusion of smaller and more homogeneous cohorts of patients and overall reduction of costs^27^.

For DBS to become fit for purpose in these large-scale endeavours, further investigations are clearly needed into the pre-analytical and analytical hurdles highlighted in this paper. This will have to include testing Nf-L blood levels in DBS from a variety of pathologies encompassing a range of potential Nf-L blood concentrations, sample preparation minimizing the confounding effect of haemoglobin and cell-membrane proteins as well as the use of techniques other than immunoassays that do not rely on the performance of target-specific antibodies.

DBS sampling using filter paper goes back to the early 1960s to facilitate collection of heel prick blood samples from newborns. While multiple examples of the potential clinical exploitation of DBS for biochemical analyses have emerged in the literature^13,28^, there has been little or no attention to the range of confounders and above all to the comparability that dry spots-based assays have with standard plasma/serum analyses for the same analyte. We believe that the need to proof-test DBS-based measurements (particularly when immunodetection is employed) using standard serum assays as benchmark and/or internal quality controls is paramount.

When whole blood dries onto the filter paper, the content of ruptured cells is released into solution only when DBS samples are reconstituted by elution, creating a highly diluted fluid contaminated by products of red cell lysis, not dissimilar to what normally recognised as haemolysed blood. As a consequence, Nf-L levels are on average 2.8 lower in DBS elutes than in plasma samples. Using the highest sensitivity platform for plasma Nf-L analysis, we note that Nf-L levels in DBS elutes show a smaller range of variability (Fig. 3B.1,B.2) compared to the higher variability of Nf-L concentrations in matched plasma samples, despite loading the same protein amount for all plasma and DBS elute samples. The possible “signal attenuation” seen in the immunodetection of Nf-L using DBS may relate to the spurious environment of the DBS elute compared to the relative purity of proteinaceous plasma/serum as previously reported^18^. The lack of correlation between plasma and matched DBS-elute samples in Nf-L expression (note the different scatter from the mean in plasma and DBS Nf-L values in Fig. 3B.1,B.2) suggests that the filter paper retention and the buffering of high concentration of haemoglobin may ultimately alter Nf-L detection. This may also explain the differences in Nf-L expression when plasma and DBS elutes are studied using WB (Fig. 4A) and ELISA-based methods (Fig. 3B.1,B.2). Haemolysis is a well-known confounder in the detection of a number of analytes, a phenomenon which may depend on the nature of the analyte and on the analytical approach used for their measurement^29^. It is important to observe that in our study, the loading of an equal protein concentration and the absence of differences of haematocrit levels (Supplementary Fig. 4) did not affect the measurement by Simoa of Nf-L in DBS elutes and the statistical differences of group separation when DBS where compared to matched plasma samples. Overall, our experiments point to three potential reasons for the lower yield and differences in DBS neurofilaments measurement compared to plasma (Fig. 3A.2-B.2): (1) the reduction of the amount of analyte that may come off the filter paper and enter solution in a form which is suitable for analysis, (2) the alteration of the biochemical structure of Nf proteins (Fig. 4) which may affect the release of Nf-L fractions transferred into solution due to the process of drying and finally, (3) the interference of haemoglobin products which may bind to or sequester the target analyte from the pool of proteins and reduce the antibody-antigen interaction in a Sandwich ELISA^30^. The detection of a relatively high amount of Nf-L in erythrocytes when compared to other blood fractions (Figs. 4B and 5A) is another indication of the importance of factoring in red cell lysis when measuring Nf-L in blood, considering that various degrees of haemolysis could affect plasma purity.

Nf-L measurement using DBS storage of blood micro-samples could also depend on protein fragmentation due to proteases activity. Proteases seem not to have a major effect on total protein recovery from DBS elute (Supplementary Fig. 1) but the different Nf-L protein expression profiles when plasma and DBS are compared by WB analysis suggest that Nf-L proteolysis is matrix-dependent (Fig. 4A). The effects of proteases on Nf derived from axonal injury and within extracellular fluids have been previously described^31^. It is possible that the proteolysis of Nf depends on the fluid microenvironment and in our case, on the matrix effect of the filter which may selectively retain protein fragments, thus altering the final composition of the Nf-L *fragmentome* and its detectability.

In this paper, we have also looked at the total protein content and at Nf-L concentration in plasma before and after spotting and eluting from dry filter paper (Figs. 2 and3B.3C.1), as previously done with other low-abundance proteins like cytokines^32–34^. The overall plasma protein profile which include Nf-L levels is reproduced in elutes from DPS, suggesting that blood micro-sampling with devices like Noviplex, which delivers plasma rather than a haemolysed fluid, could be the best option in the choice of a remote micro-sampling method. The high costs of the available DPS systems may limit their use but the likely development of more affordable DPS may soon have a significant impact on their use in large scale biomarkers studies involving the measurement of Nf-L.

## Conclusion

Here we investigate the potential of using DBS as a biological substrate for the measurement of Nf-L in the clinical stratification of ALS. Nf-L analysis in DBS elute from ALS patients and aged-matched controls is possible, but results are qualitatively and quantitatively different from those obtained from DBS-matched plasma samples. Nf-L levels in DBS elute provide discrimination between controls and ALS patients and their phenotypic variants but normalization using known loading protein concentration is needed to adjust for the chromatographic effect of retained haemoglobin in the spot elute. It is unclear whether the use of DBS may provide any useful information in situation where, unlike ALS, Nf-L blood concentration is expected to be significantly lower. Nf-L levels in plasma reconstituted from Noviplex DPS (Np-DPS) cards are much closer to those obtained testing conventional plasma samples. DPS-based Nf-L analysis could therefore be a more reliable alternative methodology to Nf-L plasma analysis.

## Material and Methods

### Participants and biological samples

Amyotrophic lateral sclerosis (ALS) patients and healthy individuals were selected from the ALS biomarkers study biorepository. Informed consent was obtained from all participants. All research was performed in accordance with relevant regulations (Ethical approval: London and the City Research Ethics Committee 1 09/H0703/27). The ALS Functional Rating Scale-Revised (ALSFRS-R; range 1 to 48, increasing levels of neurological impairment with lower scores) was used to define the level of neurological involvement and the speed of disease progression. Progression rate to last visit (PRL) was calculated as “48 - ALSFRS-R at the last visit, divided for disease duration from onset to the last visit in months” (fast ALS progression: PRL > 0.9; slow ALS progression: PRL ≤ 0.6)^34^.

### Sample collection and processing

Blood was collected by venepuncture in EDTA bottles. Drops of whole blood (approximately 80ul) were spotted onto dry blood spots (DBS), 5 spots for a total of 400 μl per card (#10534612; Whatman #903, GE Healthcare, Piscataway, NJ) and plasma was immediately recovered from the remaining whole blood sample by centrifugation. DBSs were dried at room temperature (cards have a flap to cover the sample once it is dry), then placed in zip-lock bags containing a silica desiccant. For each participants/blood sample, one card was kept at RT, one stored at −80 °C and one air-dried and stored at −80 °C. Matched plasma samples were stored at −80 °C. Dry plasma spots (DPS) were also obtained by 1) spotting drops (80 μl) of plasma obtained by centrifugation onto protein saver cards (PSC) as described above or 2) spotting blood drops onto Noviplex Card Duo (Np-DPS), (two drops of blood on each card, https://www.shimadzu.co.uk/noviplex-cards-1), a newly developed micro-sampling tool used to collect volumetric samples of plasma. Noviplex plasma spots were kept at RT in zip-lock bags containing a silica desiccant. DBS and DPS were cut at a 6 mm radius using a Uni-Core round punch-tool. The puncher and cutting mats were cleaned and sterilized after each punch. PSCs kept at −80 °C, were taken from the freezer and kept on dry-ice until punch took place. Np-DBS discs were obtained by peeling off the top layer of the Noviplex Card Duo and kept at RT in their foil pouch.

### Elution conditions and total protein recovery

DBS and DPS discs were placed into a 96-well ELISA plate containing 60 µL PBS-elution buffer per well and kept in constant shaking. Protein recovery was tested using the Pierce method (Pierce™ BCA Protein Assay Kit). The following conditions were tested for optimal protein recovery: (1) overnight incubation at 4 °C or 37 °C, one hour, (2) PH range 4.5–7.4 3) Addition to elution buffer of 0.05% Tween-20 and the effects of 1:50 protease inhibitor (P8340-Sigma).

Additional centrifugation steps were applied to maximize the yield of protein recovery and improve the elute purity from products of haemolysis: discs were removed from the 96-well plate and placed into 0.5 mL Eppendorf without cap and open at the bottom, which was then slotted inside a bigger 1.5 mL Eppendorf. The combined Eppendorfs were spun down at 10 g for 3 min and the liquid content was recovered from the bottom of the larger Eppendorf (10 to 20 µl) and put back into the corresponding well. Total DBS elutes from the 96 wells were placed into new 1.5 mL Eppendorf tubes and spun down at 20 g for 6 minutes to eliminate the erythrocyte lysate contaminant.

Noviplex DPS disc elution was performed using 100 µl of loading buffer in use for the Simoa neurofilament analysis (sample buffer diluent included into the Simoa Nf-L Advantage Kit-102258, Quanterix).

Red blood cell lysate was obtained using distilled water.

### Protein quantification methods

Pierce and Bradford assay were used for protein quantification using a bovine serum albumin (BSA) internal calibrator as reported by the manifacturer (Pierce™ BCA Protein Assay Kit - 23227- Thermo scientific; Bio-Rad Protein Assay Kit II Bio-Rad - cat. 5000002). Gel-based separation was used to evaluate qualitatively and quantitatively total protein content in elutes from DBS, DPS and dry serum spots (DSS) and band profiles were compared across samples. DBS, DPS and DSS elutes were diluted 1:60 at different PHs to study the protein content by Pierce or Bradford methods.

### Nf Western Blot analysis

100 micrograms of total protein from plasma, DBS elutes and red blood cell lysate mixed with Laemmli buffer were loaded onto 10% acrylamide gels with a marker (SeeBlue® Plus2 Cat. PLC5925). After electrophoresis, proteins were transferred onto nitrocellulose membranes and blocked with Tris buffered saline 0.1% Tween (TBS-T) containing 5% non-fat dry milk powder and placed at −20 for 1 h at room temperature. Membranes were then incubated overnight with Anti-Nf-L antibody (clone EP675Y, rabbit monoclonal-Millipore). Enhanced chemiluminescence (ECL kit; GE Healthcare), the ChemiDoc XRS + imaging system and the image lab 5.2.1 (Bio-Rad) software were used for signal detection acquisition and analysis.

### Nf measurement by Meso Scale Discovery (MSD) and Single Molecular Array (Simoa)

ALS-Fast/Slow (n: 19) and healthy controls (HC; n: 14) were analysed using an electrochemiluminescence (ECL) assay. Antibodies and assays conditions have been described in a previous publication^6^. Nf-L analysis by MSD was performed loading 50 µl of both plasma and DBS elute. For DBS analysis we considered the ratio Nf-L/haematocrit (hct). Hct were was obtained in the blood samples from where DBS were sourced. Nf analysis by single molecule array (Simoa) was performed on the same samples tested by MSD, using a commercially available Simoa Protocol for Nf-L analysis (Simoa Nf-L Advantage Kit-102258, Quanterix). An equal protein concentration (10 µg/µl) was loaded for all samples in study. The Simoa platform was used to measure Nf-L from Np-DPS. In this case, a mix of n.13 HC, neurological controls and Fast/Slow ALS were used. Nf-L analysis was performed using Np-DPS elute, plasma and diluted plasma (7 µl of plasma were diluted with Simoa diluent buffer to a final volume of 100 µl) to simulate Np-DPS elute analysis conditions. The same platform was used for the measurement of Nf-L in Red blood cells and matched plasma. Unlike for the DBS study where equal loading protein concentration was used, for the measurements of Nf-L in Np-DBS, red blood cells and matched plasma, an equal loading volume was utilised.

### Statistical analysis

Graph Prism v.7 was used for statistical analysis. The Passing–Bablok regression analysis was utilised to determine the correlation between DBS elute and plasma Nf-L levels measured by MSD. Comparison of protein expression values from DBS/Np-DPS elutes and plasma was performed using Pearson’s correlation analysis, while group analysis was obtained using the multiple comparisons Kruskal-Wallis test. Adjustment for age was also undertaken with 1) univariate general linear model with covariate of age (SPSS v25) and 2) calculating the expected Nf-L level in each control adding the amount of Nf-L that would be expected factoring in additional 9 years of age, using the function reported by Siller *et al*., where a 2.2% increase of Nf-L is reported for each additional year of age. Together with the adjustment for age performed in the group analysis, this method provides an additional way to normalize for age differences between the groups based on age-related increase of blood Nf-L levels. Evaluation of diagnostic performance was undertaken using a Receiving Operator Characteristic (ROC) analysis in Graph Prism v.7, comparing plasma and DBS elutes from ALS patients and control individuals.

## Supplementary information

Supplementary Information 
