# Author Correction: The potential of neurofilaments analysis using dry-blood and plasma spots

**DOI:** 10.1038/s41598-020-67914-6

**Published:** 2020-06-26

**Authors:** Vittoria Lombardi, Daniele Carassiti, Gavin Giovannoni, Ching-Hua Lu, Rocco Adiutori, Andrea Malaspina

**Affiliations:** 1 Blizard Institute, Queen Mary, University of London, London, UK; 2 School of Medicine, China Medical University, Taichung, Taiwan

Correction to: *Scientific Reports* 10.1038/s41598-019-54310-y, published online 09 January 2020

The Acknowledgements section in this Article is incomplete.

“The authors would like to thank CReATE and the ALS Association (US) for the award of the grant supporting the study: “Going Dry: empowering neurofilament-based biomarkers studies for disease monitoring in amyotrophic lateral sclerosis”. We would also like to thank all ALS patients and their families for their support to this research project and we acknowledge the MND Association (UK) for their help in establishing the ALS biomarkers biobank and contributing with significant resources in the sample collection phase and to general aspect of research in ALS. We also acknowledge the support of the North-Thames Local Research Network (London) in the recruitment process and in the collection and processing of biological samples.”

should read:

“The authors would like to thank CReATe and the ALS Association (US) for the award of the grant supporting the study: “Going Dry: empowering neurofilament-based biomarkers studies for disease monitoring in amyotrophic lateral sclerosis”. The CReATe consortium (U54NS092091) is part of the Rare Diseases Clinical Research Network (RDCRN), an initiative of the Office of Rare Diseases Research (ORDR), NCATS. CReATe is funded through a collaboration between NCATS, and the NINDS. We would also like to thank all ALS patients and their families for their support to this research project and we acknowledge the MND Association (UK) for their help in establishing the ALS biomarkers biobank and contributing with significant resources in the sample collection phase and to general aspect of research in ALS. We also acknowledge the support of the North-Thames Local Research Network (London) in the recruitment process and in the collection and processing of biological samples.”

