# Supplementary material for: The potential of neurofilaments analysis using dry-blood and plasma spots
Source: Sci Rep. 2020 Jan 9;10:97. doi: 10.1038/s41598-019-54310-y (PMC6952412; doi:10.1038/s41598-019-54310-y)
Supplement: Supplementary file 1 — Supplementary Information [file 41598_2019_54310_MOESM1_ESM.pdf]

The potential of neurofilaments analysis using dry-blood and plasma spots

Vittoria Lombardi, Daniele Carassiti, Gavin Giovannoni, Ching-Hua Lu, Rocco Adiutori, Andrea Malaspina

SUPPLEMENTARY DATA

Figure 1

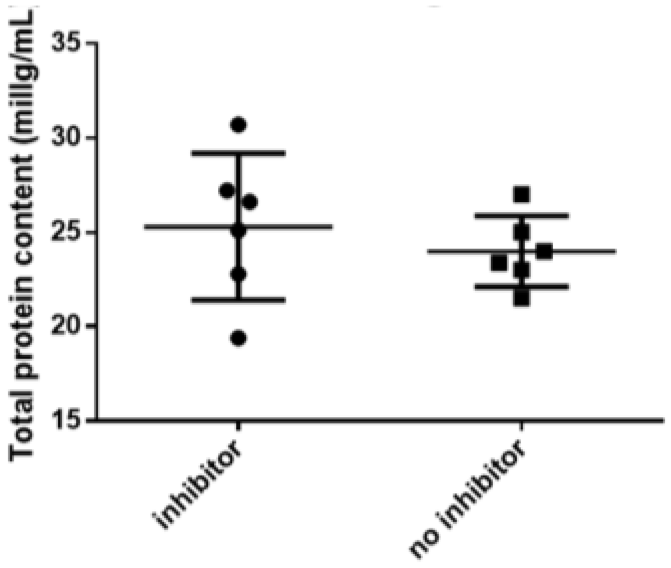

Figure 1 Total protein content in DBS elute of 6 controls with and without proteases inhibitor after 24 hours from the elution. Non-significant differences with t-test (Mann-Whitney test).

Figure 2

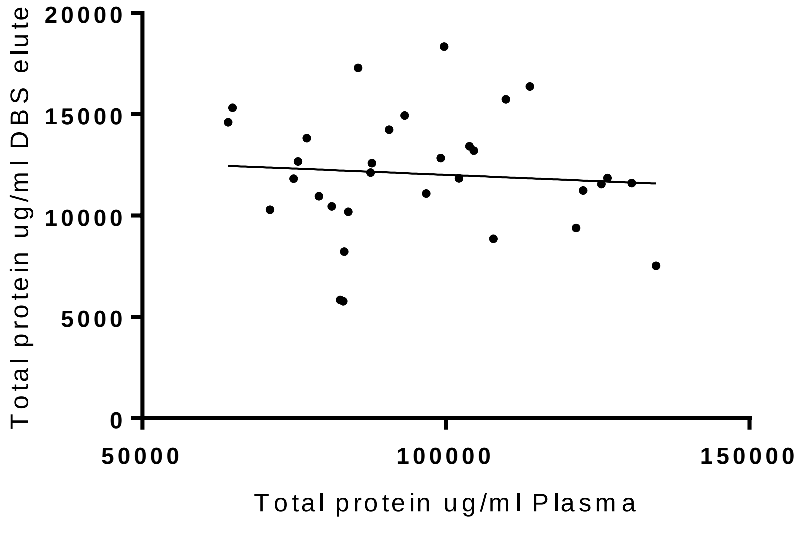

Figure 2 Total protein correlation between Plasma and DBS elute. Non-significant correlation, Pearson analysis, p=0.6.

Figure 3

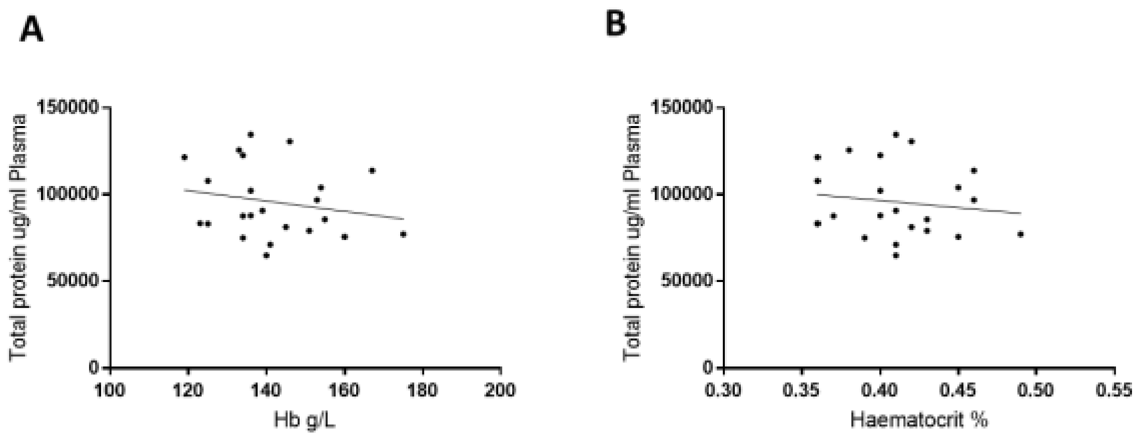

Figure 3 Correlation between Hb/Haematocrit and Total protein in plasma. Non-significant correlation, Pearson analysis. A: p=0.26; B: p=0.4

Figure 4

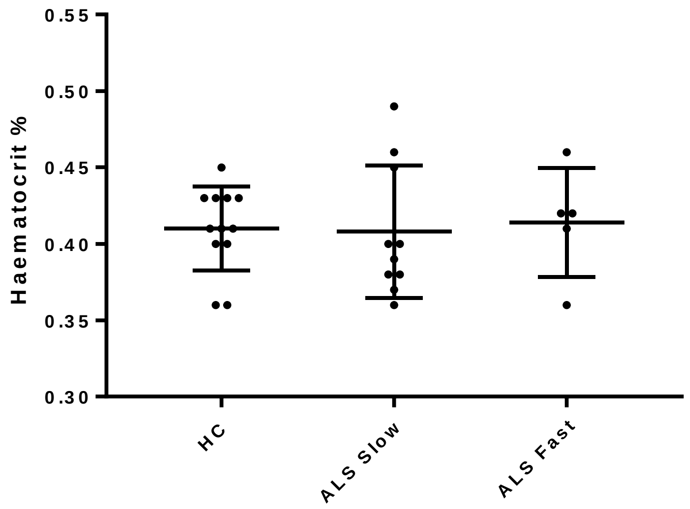

Figure 4 Haematocrit % in HC, slow and fast ALS patients. Non-significant differences with one-way ANOVA test, p> 0, 99.

SOURCE GEL IMAGES

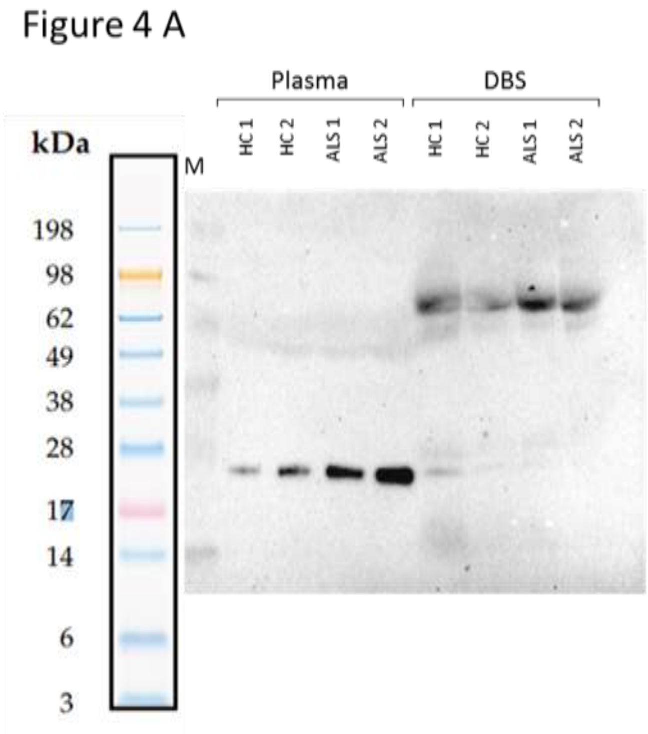

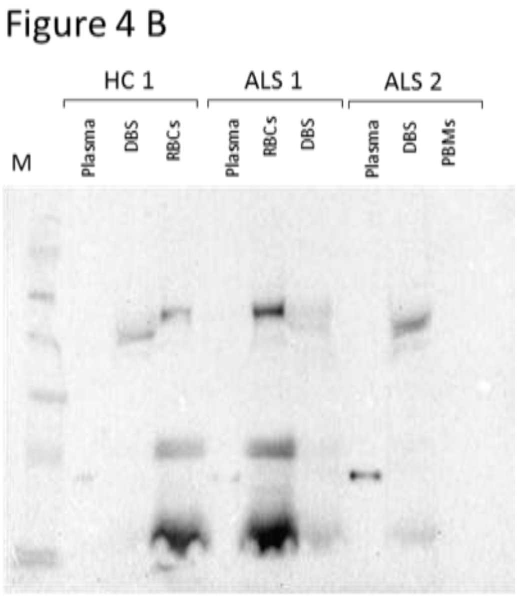

Legend: western blot analysis of neurofilament light (NF-L) comparing plasma and elute from DBS obtained from the same blood samples of healthy controls (plasma HC 1, 2; DBS HC 1, 2) and ALS individuals (plasma ALS 1, 2; DBS ALS 1, 2 - Figure 4 A). NF-L expression is also shown in plasma, DBS elutes, red blood cells (RBCs) and polymorphonuclear cells (PBMCs) obtained from blood samples of the same control and ALS individuals (HC1 and ALS 1 and 2 - Figure 4B). In the manuscript, only the gel lanes where ALS1 blood products were loaded are reported in Figure 4 B.

Marker (M): SeeBlue® Plus2 Cat. PLC5925

<u>Figure 4 A</u>

ChemiDoc XRS+ imaging system
Software: Image Lab 5.2.1
Exposure: 122 sec

<u>Figure 4 B</u>

ChemiDoc XRS+ imaging system
Software: Image Lab 5.2.1
Exposure: 412.4 sec
